# Market policy as an innovative element of marketing in the Romanian healthcare services – an approach focused on the patient

**Published:** 2015

**Authors:** BI Coculescu, EC Coculescu, A Radu, L Petrescu, VL Purcărea

**Affiliations:** * “Titu Maiorescu” University, Faculty of Medicine; Centre of Preventive Medicine, Ministry of National Defence, Bucharest, Romania; ** “Carol Davila” University of Medicine and Pharmacy, Faculty of Dental Medicine, Bucharest, Romania; *** “Carol Davila” University of Medicine and Pharmacy, Faculty of Medicine, Bucharest, Romania

**Keywords:** Romanian health system, healthcare marketing, marketing policy

## Abstract

The orientation towards one of the marketing policies with a major impact in organizations providing healthcare services, requires a careful analysis of the needs and aspirations of customers, targeting those patients whose needs the service organization can achieve through the existing resources at the respective health facility, finding the most effective way of achieving benefits associated with reduced costs to maximizing profits, placing the offers for medical services required by the patients on the market, as well as promptly reacting and acting to the changes of health services market which is constantly evolving through a flexible organizing and functioning structure, connected to the financial needs of the patients.

## Introduction

In order to strengthen its position in the healthcare system and cope with the difficulties caused by the competitive environment of other health institutions, state and private medical organizations require appropriate management policies. These instruments of the health services market seek, among other things, to focus efforts and facilitate coordination activities, to efficiently use the material resources, reducing the uncertainty of the expected results and promoting efficiency. Moreover, the key terms of the success of management strategies implemented by a medical organization are managerial vision, personal values, and organizational culture [**1**-**3**].

The marketing strategy can be defined as the set of principles, policies, and procedures guiding the marketing of the firm on its target market. It is based on three elements: budget (generally corresponding to a percentage of the turnover and twice the segment market hoped to achieve), marketing-mix (which integrates all the available variables of the organization to influence the target market, McCarthy’s “four Ps”, respectively), and the distribution procedure (the manner in which the budget will be distributed to different products, customer segments and sale territories in an effort to maximize the return of marketing investments on a long term) [**4**].

Kotler identified five distinct stages of the implementation of marketing strategies:

1) marketing is advertisements, promotions and advertising; 2) marketing is when you smile and have a friendly atmosphere around; 3) marketing is innovation; 4) marketing is market positioning and 5) marketing is analysis, planning and control [**5**].

An important task for any medical organization is the way it manages to promote the benefits of its services so that they are received by the patients as answers to their needs. Specifically, the strategy targeting the practical problems can be done through a clear and concise action plan by knowing the potential target patients and their expectations. Strategy pays off only if supported by healthcare services users.

The final step in adopting a strategy consists, otherwise, of sharing innovative solutions from the marketing department to the beneficiaries, hence the necessity of knowing their profile.

## Discussion

In order to implement the marketing strategy, the manager of the medical organization must ensure working interrelationships between the various departments of the organization, stimulate the activity of the staff, create information systems, and control the administrative processes. Simultaneously, he/ she must constantly assess attitudes and decisions, have a good self-evaluation and a precise vision of the health organization’s social involvement.

Due to the implementation of policies/ strategies, marketing, healthcare organizations are on the one hand, thanks to new information technologies and new media, in the situation of being up to date in an attempt to keep pace with an accelerated changing market and position themselves ahead of the competition, on the other hand, because improving methods and marketing tools, they create a feeling of superiority in terms of the quality of services.

That is why we must re-imagine every facet of the way we act by arming ourselves with education, and entrepreneurial spirit to cope with a tough economic competition which is given for the best professional occupations in which people must re-imagine themselves with spirit, energy, passion, and talent [**4**].

 Porter and Teisberg (2006) argue that:

• establishing the tasks to be delivered is probably the fundamental strategy for each supplier (the latter must choose the set of medical conditions that can achieve true excellence in terms of value for patients, particularly taking into account the patient’s mix, skills and other circumstances; in addition, the supplier must decide what role to play in the health care cycle and what services to provide to ensure the overall results for the patient);

• a part of the strategic choice of services consists in matching the complexity and acuity of the conditions diagnosed and treating them skillfully (if you cannot provide routine or simple services at a competitive price, then do not give them; if you do not have the expertise and capabilities to provide excellent results, do not provide complex and non-usual services) [**6**-**8**].

The basic element in the organization of health services is the capitalization of products and services that create uniqueness, which implies a change of mentality in the treatment of patients from health care providers.

Marketing strategy for health care services is essentially represented by the position and conduct of the medical organization towards the business environment, and, at the same time, its attitude in relation to the institution’s own components. A higher position in the strategic approach to medical marketing activity and adaptation to environmental organization consists in market segmentation, target market choice, market positioning, and elaboration of the marketing strategy to achieve the objectives.

**Fig. 1:**
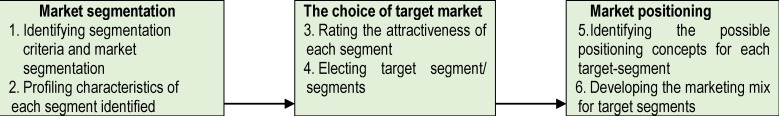
Stages of the process of adapting the organization to the market environment [**4**,**5**]

Therefore, market segmentation leads to a more accurate positioning of the organization within the business environment, an important step for a better adjustment to the financial environment, *followed de facto* by other actions subsequent to segmentation, namely, the choice of market-targets and then market positioning (**Fig. 1**).

It is important to distinguish between consumer marketing and services marketing so as to increase the marketing mix, the central element of the marketing strategy, from “the 4P” to “the 7 P” (**Table 1**).

**Table 1:** The marketing mix in services - vital tool of marketing policy [**4**]

The “7P”	Marketing mix of services
1. **Product**	Traditional mix of marketing
2. **Price**	(McCarthy, 1960)
3. **Place**	
4. **Promotion**	
5. **Physical evidence**	Environment in which the service occurs and the organization enters into direct relationship, as well as any material goods that facilitate the creation or transmission of the service.
6. **Participants** - organization staff, other clients	All individuals who play a role in influencing service delivery and thus buyer perceptions, specifically, company staff and other customers in rural service.
7. **Process**	All the procedures, mechanisms, and flow of activities that create and provide the service.

This is an expanded mix, which represents a simple way of organizing, by which elements that are involved in service organizations can be studied. The expanded mix also resulted from partially solving specific elements that emerged during quality control and interaction with patients in supplying healthcare services. It highlights both the role of health professionals and of patients in medical activities, stressing the importance of marketing in both situations.

As a corollary of the above, it can be said that the expanded marketing mix, the elements which are mutually interdependent, represent putting into practice the medical marketing strategy. The elements of the marketing mix play a critical role in the success of marketing in the health care service system. The specific characteristics of health services and in particular the distribution of the product causes a series of adjustments and/ or changes in strategies related to the elements of the marketing mix.

**Table 2:** Adjustments of the marketing mix [**4**]

The marketing mix for services (Bitner, 1981)	The marketing mix for public services (Hayden, 1997)
Product	Product
	- product or service offered.
Price	Price
	- Prices for consumers;
	- Financial and other costs borne by consumers or customers.
Place	Place or access
	- Bringing the product to the consumer.
Promotion	Promotion
	- Communication with consumers about the product/ service.
Physical evidences	Customer service
Participants (staff, other clients)	- Benefits for consumers and customers;
Processes	- Non-technical and clinical aspects beyond the process itself.

The essential factor of marketing strategy in the field of health services business is the quality of services, which in turn depend on the consciousness and fairness in medical activities, training of medical staff, promptness, and correct attitude towards patients.

Achieving excellence in the health services of a medical organization is based on the establishment and adoption of a coherent policy, as well as clarifying and promoting the value system within that organization.

The notion of quality is a result of the comparison between the action of providing care services desired by the patient and the service received or, in other words, the degree to which the service provided meets the expectations. Largely, the quality of patient care is compared by the patient in relation to what he/ she wants from the respective service and the experience that he/ she has accumulated over the years regarding the medical work. When the patient’s expectations are met, objectively or subjectively, he/ she recognizes the quality of medical performance.

**Table 3:** Factors that lead customers to switching providers [**4**]

Prices	Reaction of provider to a flawed service
• High price	• Negative reaction
• Price increases	• No reaction
• Unfair rates	• Reluctant reaction
• Rates misleading	Competition
Drawbacks	• The customer found a better service
• Installation / working hours	Ethical issues
• Hold over performance program	• The supplier tries to trick the client
• Hold over performance of the contract	• The supplier has a pushy sales approach
Failure to observe basic service promise	• Risks to the personal safety and security
• Errors in the service	• Conflicts of interest
• Errors in billing service	
• Catastrophic results service	

An important role in assessing the quality of services by the patients is played by the emotional factors (impressions) and the environment (circumstances) in which health care operates.

Given that ways and means for assessing the quality of care cannot be covered entirely by the medical organization, that institution cannot influence them in a special way. The process of evaluating the quality of medical services is extremely complex and consists of: simultaneous delivery and acceptance of health services, patient involvement in the delivery process, the interrelation between the health facility contact staff and patient and links between providers of health services and patient, on the other hand.

## Conclusion

In marketing policies, health organizations must identify the multidimensional needs of patients and subsequently find and direct their resources towards the creation of services to meet these demands.

Defining quality by the way the medical unit manages to reach or exceed the needs and expectations of the patients; we can say that the quality of health services is, ultimately, the level perceived by the patient. So, in a system full of the unexpected, as the health care services system is, one thing is certain, namely that the patient appreciates quality.

In the context in which excellence in the quality of health care services, meeting the healthcare systems, the customer’s expectations are recognized as a priority for the entire population, adopting methods and marketing concepts as an alternative to identify opportunities for innovation in health care services can only be beneficial. In this new marketing philosophy that puts the patient at the center, all activities and policies are targeted to meet the expectations and needs of the patient, so that the quality of performance is more important than the maximum amount of benefits made by that organization.
